# Clinical impact of genetic testing in inherited kidney diseases

**DOI:** 10.1093/ckj/sfag160

**Published:** 2026-05-19

**Authors:** Lea M Merz, Sarah Stopp, Ilona Krey, Fabian Baalmann, Emilia Marczak, Nora Liebmann, Olga Hempel, Bastian M Krüger, Marie Engesser, Anne-Christin Teichmann, Franziska Schnabel, Rami A Jamra, Johannes Lemke, Carsten Bergmann, Tom H Lindner, Jonathan de Fallois, Johannes Münch, Jan Halbritter, Katalin Dittrich, Friederike Petzold

**Affiliations:** Department of Pediatric Nephrology, University of Leipzig Medical Center, Leipzig, Germany; Division of Nephrology, University of Leipzig Medical Center, Leipzig, Germany; Institute of Human Genetics, University of Leipzig Medical Center, Leipzig, Germany; Division of Nephrology, University of Leipzig Medical Center, Leipzig, Germany; Department of Pediatric Nephrology, University of Leipzig Medical Center, Leipzig, Germany; Department of Pediatric Nephrology, University of Leipzig Medical Center, Leipzig, Germany; Department of Pediatric Nephrology, University of Leipzig Medical Center, Leipzig, Germany; Division of Nephrology, University of Leipzig Medical Center, Leipzig, Germany; Division of Nephrology, University of Leipzig Medical Center, Leipzig, Germany; Institute of Human Genetics, University of Leipzig Medical Center, Leipzig, Germany; Institute of Human Genetics, University of Leipzig Medical Center, Leipzig, Germany; Institute of Human Genetics, University of Leipzig Medical Center, Leipzig, Germany; Institute of Human Genetics, University of Leipzig Medical Center, Leipzig, Germany; Medizinische Genetik Mainz, Limbach Genetics, Mainz, Germany; Division of Nephrology, University of Leipzig Medical Center, Leipzig, Germany; Division of Nephrology, University of Leipzig Medical Center, Leipzig, Germany; Division of Nephrology, University of Leipzig Medical Center, Leipzig, Germany; Medical Clinic for Nephrology, Hypertensiology, Kidney Transplantation, Endocrinology and Pneumology, University Medicine Mannheim, Mannheim, Germany; Division of Nephrology, University of Leipzig Medical Center, Leipzig, Germany; Department of Nephrology and Medical Intensive Care, Charité-Universitätsmedizin Berlin, Corporate Member of Freie Universität Berlin and Humboldt-Universität zu Berlin, Berlin, Germany; Department of Pediatric Nephrology, University of Leipzig Medical Center, Leipzig, Germany; Division of Nephrology, University of Leipzig Medical Center, Leipzig, Germany

**Keywords:** ADPKD, chronic renal failure, FSGS, nephrotic syndrome, pediatrics, prognosis

## Abstract

**Background:**

Inherited kidney diseases (IKDs) and congenital anomalies of the kidney and urinary tract (CAKUT) are a clinically heterogeneous subset of chronic kidney disease and rank among the leading causes of kidney failure (KF), especially in younger patients. Although next-generation sequencing has expanded diagnostic opportunities, data linking genetic findings to clinical outcomes across both adults and children remain limited.

**Methods:**

We enrolled 256 patients with suspected IKD (175 adults, 81 children) at a single tertiary center between 2020 and 2023. Genetic testing was performed using targeted panels or exome sequencing, including copy number variant (CNV) and *MUC1* analyses. Clinical data, including family history, proteinuria, hematuria, extrarenal manifestations, and kidney survival, were systematically assessed.

**Results:**

Pathogenic variants were identified in 38.7% of patients. Five genes (*PKD1, PKD2, COL4A5, COL4A4*, and *HNF1B*) accounted for 62.6% of solved cases. Diagnostic yield was highest in cystic kidney diseases (72.0%), followed by tubulopathies (34.3%), glomerulopathies (25.8%), and CAKUT (19.2%). Multivariable regression identified positive family history, extrarenal manifestations, and arterial hypertension as predictors of a genetic diagnosis, with tubular proteinuria serving as an additional predictor in children. Kidney survival varied substantially across genetic subgroups: patients with *PKD2* variants and tubulopathies showed more favorable outcomes, whereas individuals with *COL4A5*-associated Alport syndrome and glomerulopathies progressed rapidly. In the overall cohort, females reached 50% KF significantly later than males. Genetically solved men had the poorest outcomes, with a significant difference compared with solved women; however, this difference was reduced to a nonsignificant trend after excluding X-linked disorders.

**Conclusions:**

Genetic testing provided clinically relevant diagnoses in nearly 40% of patients with suspected IKD, enabling more accurate prognostication and patient stratification. High yield in cystic disease and pediatric tubular proteinuria, sex-specific survival differences, and CNV analysis highlight the value of integrating genetics into routine nephrology care to guide diagnosis, management, and family counselling.

## Graphical Abstract

**Graphical Abstract:**
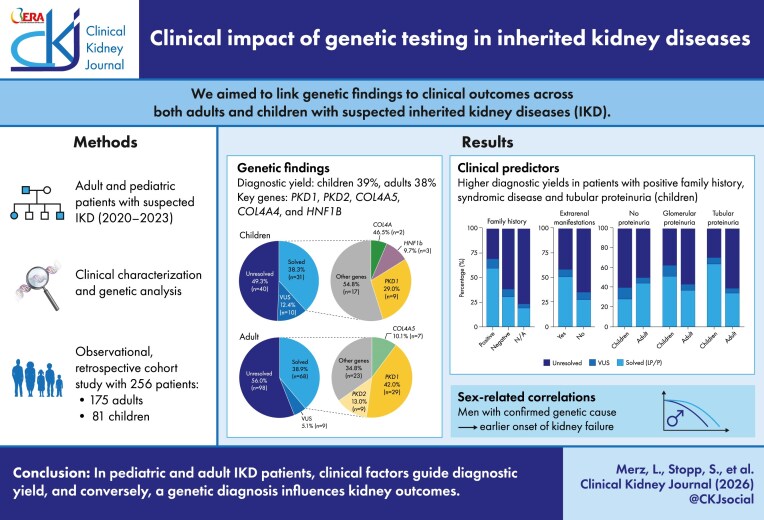

KEY LEARNING POINTS **What was known:**

- Inherited kidney diseases (IKD) account for 10%–15% of patients reaching kidney replacement therapy.
- Genetic testing yields 20%–50% across studies; most diagnoses involve five core genes (*PKD1, PKD2, COL4A5, COL4A4, HNF1B*).
- The highest yield is in cystic kidney disease, while kidney outcome is most favorable in tubulopathies.

**This study adds:**

- In children, tubular proteinuria was the strongest predictor of a genetic diagnosis, while hematuria did not increase yield in glomerular disease.
- IKD show age-specific diagnostic windows: CAKUT almost exclusively in childhood, cystic kidney disease at 40–60 years, glomerulopathies with bimodal peaks. CNV analysis raised yield in pediatric CAKUT (13.9%) and identified a novel CNV with a severe phenotype.
- Solved women showed better renal survival than solved men, but this difference became nonsignificant after excluding X-linked cases. Kidney outcomes were also worse in solved cases of CAKUT, glomerulopathies, and cystic kidney disease compared with unsolved cases.

**Potential impact:**

- Prioritizing genetic testing in suspected IKD, particularly with tubular proteinuria, increases diagnostic yield; adding CNV analysis improves detection in pediatric CAKUT and reveals novel pathogenic variants.
- Genetically solved patients and men show worse kidney survival, supporting risk-adapted follow-up and early management strategies.
- Age-specific diagnostic windows for CAKUT, cystic kidney disease, and glomerulopathies need recognition and discussion to refine the timing of genetic testing, e.g. earlier diagnostics in childhood or limited utility in adult CAKUT patients.

## INTRODUCTION

Chronic kidney disease (CKD), characterized by progressive loss of kidney function, affects 10%–15% of the global population [1, 2] and represents a major public health burden [1, 2]. Inherited kidney diseases (IKDs), defined as monogenic disorders directly causing kidney dysfunction, contribute substantially to advanced CKD, particularly in children and young adults [3, 4]. Although individually rare, IKDs account for 10%–15% of adults receiving kidney replacement therapy (KRT), with even higher prevalence in pediatric populations [4]. IKDs and congenital anomalies of the kidney and urinary tract (CAKUT) together account for 8.9% (IKD 7.4%, CAKUT 1.5%) of kidney failure (KF) among patients on KRT, ranking fourth behind diabetes, hypertension, and glomerulonephritis [4]. They are the most common cause of KF among patients under 20 years of age, responsible for 41.0% of cases [4].

The most frequent IKDs are cystic and glomerular nephropathies, with autosomal dominant polycystic kidney disease (ADPKD, MIM #173900) and Alport syndrome (AS, MIM #301050) predominating [5, 6]. Cystic kidney diseases are typically caused by pathogenic *PKD1, PKD2*, and *PKHD1* variants and characterized by a broad clinical spectrum, potentially being phenocopied by other cystic entities such as nephronophthisis (MIM #256100) and *HNF1B*-related disease (MIM #137920). AS results from pathogenic variants in *COL4A5* (X-linked), *COL4A3*, or *COL4A4*, with clinical manifestations ranging from isolated microhematuria to proteinuria and progressive KF. In glomerular nephropathies, steroid-resistant nephrotic syndrome, is linked to *NPHS2, NPHS1*, and *WT1* alterations and represents a major cause of early KF [7, 8]. In CAKUT, ∼50 monogenic and 135 syndromic genes have been identified [9, 10], although a monogenic cause is found in only 5%–20% of cases [9].

Determining the underlying cause of IKD is essential for accurate prognosis and effective management. However, evidence remains limited: only a handful of cohort studies have quantified the diagnostic yield of genetic testing cohorts with suspected IKD [11–14]. While highlighting a broad genetic spectrum, robust clinical data in genetically defined IKD cohorts are lacking. In particular, studies linking genetic findings to clinical trajectories–such as sex-specific differences and clinical predictors of genetic findings––are scarce [11–14]. This study evaluates diagnostic yield and clinical spectrum in 256 patients (175 adults, 81 children) with suspected IKD, focusing on clinical predictors, such as proteinuria, hematuria, extrarenal manifestations, and exploring kidney survival, and genetic findings across age and sex.

## MATERIALS AND METHODS

### Patient population and selection

Adult and pediatric patients with suspected IKD were recruited from the University Hospital Leipzig Nephrology Department (2020–23, *n* = 256). Ethics approval was obtained (224/16-ek, 402/16-ek), and written informed consent was provided.

Baseline assessments included DNA sampling, kidney ultrasound, family history, physical examination for extrarenal features, and laboratory testing (serum creatinine, urea, cystatin C, eGFR, hematuria, proteinuria). eGFR was calculated using the CKD-EPI equation for adults and the Schwartz 2009 formula for children. In children, arterial hypertension was defined as blood pressure ≥95th percentile for age, sex, and height according to the 2017 American Academy of Pediatrics guidelines, consistent with German reference percentiles [15, 16]. In adults, hypertension was defined as a systolic blood pressure ≥140 mmHg and/or a diastolic blood pressure ≥90 mmHg, based on repeated office measurements, in accordance with the European Society of Hypertension guidelines [17]. Glomerular proteinuria was defined as albuminuria ≥A2 (≥30 mg/g creatinine) or nonselective proteinuria per KDIGO guidelines. Tubular proteinuria was defined as an elevated urinary α1-microglobulin-to-creatinine ratio (mg/g) and considered present at values >14 mg/g. When a disease-causing variant linked to a syndromic phenotype was detected, reverse phenotyping was performed, including targeted examination, subspecialty referrals, and imaging. Segregation analysis was conducted when possible. Based on clinical and molecular findings, patients were assigned to cystic kidney diseases, glomerulopathies, tubulopathies, or CAKUT. Patients were classified as having CKD of unknown origin (CKDx) when comprehensive assessment, including phenotypic evaluation, laboratory testing, imaging, histopathology, and genetic analysis, did not identify a specific cause of CKD [15].

### Genetic testing

Blood samples were obtained for genetic analysis using either the TruSight One panel or the TWIST Exome 2.0 platform. In the adult cohort, targeted gene panels were primarily used (*n* = 131/175, 74.9%, Supplementary Table 1), whereas most pediatric cases underwent exome sequencing (ES, *n* = 66/81, 81.5%, Supplementary Table S1). Virtual panels were first applied to the exome data; if results were negative, comprehensive re-analysis of the full exome was performed. Karyotyping was performed in all pediatric patients with congenital malformations and a syndromic phenotype. Copy number variant (CNV) analysis was systematically performed in the entire cohort, irrespective of sequencing approach. *MUC1* was systematically evaluated in all patients using the VNtyper tool [18, 19].

Detected single-nucleotide variants and CNVs were classified using ACMG-AMP (Genomics-Association for Molecular Pathology) and ClinGen criteria [20–22]. Variants were classified according to the standard gene variant nomenclature [23]. Using the HGMD database (HGMD Professional 2024.4) and ClinVar [24], it was assessed whether each variant had been previously reported [23]. Conventional karyotyping was performed according to standard protocols in one proband with a complex CNV.

### Statistics

Statistical analyses were performed using GraphPad Prism (version 10.1.1). Unless otherwise specified, nonparametric tests (Mann–Whitney U or Kruskal–Wallis) were applied. Statistical significance was defined as *P* < .05 (*P < .01; **P < .001; ***P < .0001; ns = not significant). To identify independent predictors of obtaining a genetic diagnosis, we performed multivariable logistic regression using GraphPad Prism. The following variables were included: age, family history, extrarenal manifestations, arterial hypertension, baseline eGFR, sex, proteinuria subtype, hematuria, and recurrent urinary tract infections. To address potential confounding by ADPKD, two models were constructed: (1) a primary model including ADPKD status as an adjustment variable, and (2) a secondary model excluding ADPKD patients to evaluate predictors within the non-ADPKD subgroup. Categorical variables were coded using clinically meaningful reference categories (e.g. absence vs presence for binary variables and positive family history as reference). Odds ratios (ORs) with 95% confidence intervals (CIs) were calculated.

## RESULTS

### Clinical characteristics

The median age at genetic testing was 4.0 years (IQR 0.0–11.0) in the pediatric and 41.0 years (IQR 33.0–53.0) in the adult cohort (Table 1). The cohort consisted of equal proportions of males and females (48.4% male, *n* = 124/256; 51.2% female, *n* = 131/256). About 37% of participants in both cohorts had a positive family history; however, particularly in the adult cohort, family history was not available in 36.6% of cases (*n* = 64/175, classified as NA, Table 1). In 5 out of 256 families (1.9%), consanguinity was reported. Relevant hematuria was observed in 16.0% of children (*n* = 13/81) and 22.3% of adults (*n* = 39/175). Glomerular proteinuria was present in 42.0% of the pediatric patients (*n* = 34/81), compared to 52.6% of adults (*n* = 92/175). Conversely, tubular proteinuria was diagnosed in 32.0% of adult patients (*n* = 56/175), while it was noted in 21.0% of children (*n* = 17/81). Extrarenal manifestations were observed in 46.9% (*n* = 120/256; adults: 84/175; children: 36/81). In 25.8% KF was present (*n* = 66/256; adults: 56/175; children: 10/81), including 17.2% (*n* = 44/256; adults: 39/44; children: 5/44) currently on dialysis and 8.6% (*n* = 22/256; adults: 17/175; children: 5/81) with a sustained functioning kidney transplant at the time of genetic testing (Table 1).

**Table 1:** Clinical characteristics of our pediatric and adult cohort.

		Pediatric cohort	Adult cohort	Total cohort
Age at genetic testing		4.0 (IQR 0.0–11.0)	41.0 (IQR 33.0–53.0)	34.0 (14.3–47.0)
Sex		m: 50.6% (*n* = 41)	m: 47.4% (*n* = 83)	m: 48.4% (*n* = 124)
		f: 48.1% (*n* = 39)	f: 52.6% (*n* = 92)	f: 51.2% (*n* = 131)
Positive family history (y/n)		Positive: 38.3% (*n* = 31)	Positive: 36.6% (*n* = 64)	Positive: 37.1% (*n* = 95)
		Negative: 53.1% (*n* = 43)	Negative: 26.8% (*n* = 47)	Negative: 35.2% (*n* = 90)
		NA: 8.6% (*n* = 7)	NA: 36.6% (*n* = 64)	NA: 27.7% (*n* = 71)
Extrarenal manifestation		Yes: 44.4% (*n* = 36)	Yes: 48.0% (*n* = 84)	Yes: 46.9% (*n* = 120)
		No: 51.9% (*n* = 42)	No: 50.9% (*n* = 89)	No: 51.2% (*n* = 131)
		NA: 3.7% (*n* = 3)	NA: 1.1% (*n* = 2)	NA: 1.9% (*n* = 5)
Kidney function	CKD stage 1–2	25.9% (*n* = 21)	43.4% (*n* = 76)	37.9% (*n* = 97)
	CKD stage 3–4	8.6% (*n* = 7)	22.3% (*n* = 39)	18.0% (*n* = 46)
	CKD stage 5, KF	12.3% (*n* = 10)	32.0% (*n* = 56)	25.8% (*n* = 66)
	KTx	6.1% (*n* = 5)	9.7% (*n* = 17)	8.6% (*n* = 22)
Urinary findings	Hematuria (y/n)	y: 16.0% (*n* = 13)	y: 22.3% (*n* = 39)	y: 20.3% (*n* = 52)
		n: 82.7% (*n* = 67)	n: 66.3% (*n* = 116)	n: 71.5% (*n* = 183)
		NA: 1.2% (*n* = 1)	NA: 11.4% (*n* = 20)	NA: 8.2% (*n* = 21)
	Glomerular Proteinuria	43.2% (*n* = 35)	54.3% (*n* = 95)	50.8% (*n* = 130)
	Tubular Proteinuria	21.0% (*n* = 17)	32.0% (*n* = 56)	28.5% (*n* = 73)
Clinical disease groups	Cystic Kidney Diseases	28.4% (*n* = 23)	33.7% (*n* = 59)	32.0% (*n* = 82)
	Glomerulopathies	19.8% (*n* = 16)	28.6% (*n* = 50)	25.8% (*n* = 66)
	Tubulopathies	7.4% (*n* = 6)	16.6% (*n* = 29)	13.7% (*n* = 35)
	CAKUT	44.4% (*n* = 36)	9.1% (*n* = 16)	20.3% (*n* = 52)
	CKD of Unknown Origin (CKDx)	0.0% (*n* = 0)	12.0% (*n* = 21)	8.2% (*n* = 21)

### Genetic testing identified disease-causing variants in 38.7% of the Total cohort

**Figure 1:**
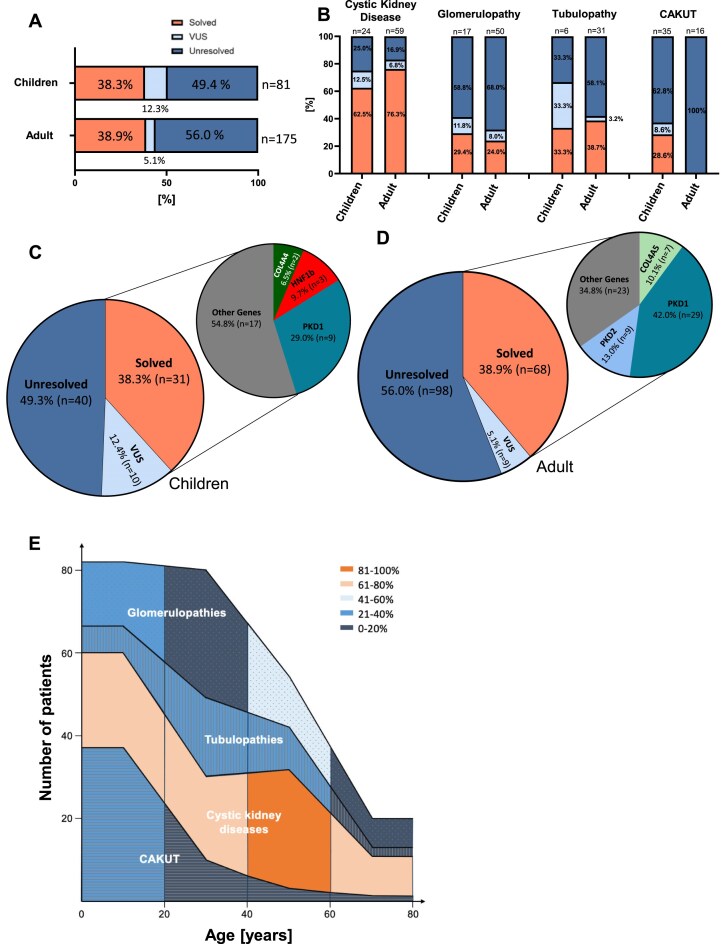
Diagnostic yield and genetic spectrum in inherited kidney disease. (A) Proportion of cases harboring pathogenic variants, variants of uncertain significance (VUS), or remaining unsolved.(B) Diagnostic yield across clinical disease subgroups. Genetic spectrum in pediatric (C) and adult cohort (D). (E) Age-dependent diagnostic testing of the clinical disease subgroups.

We performed genetic analysis in 256 patients with suspected IKD and identified disease-causing variants in 38.7% of cases (*n* = 99/256). Detailed genotypic and phenotypic information, including full HGVS variant annotation, zygosity, ACMG classification, kidney phenotype, and extrarenal symptoms for all 99 genetically solved cases, is provided in Supplementary Table S2. A comparison of clinical characteristics between solved and unsolved cases is shown in Supplementary Table S3. The diagnostic yield was comparable between children and adults, with disease-causing variants detected in 38.3% of pediatric patients (*n* = 31/81) and 38.9% of adults (*n* = 68/175, *P* > .9999; ns, Fig. 1A). In the overall cohort, patients with cystic kidney diseases showed the highest diagnostic yield at 72.0% (*n* = 59/82). This was followed by tubulopathies, with a diagnostic yield of 34.3% (*n* = 12/35, *P* = .0013, **), and glomerulopathies, with 25.8% (*n* = 17/66, *P* < .0001, ***, Fig. 1B). Detection rate was lower for patients with CAKUT at 19.2% (*n* = 10/52, *P* < .0001, ***, Fig. 1B). Of the 30 patients initially referred with chronic kidney disease of unknown etiology (CKDx), 30% (*n* = 9/30) received a genetic diagnosis. The most frequently identified genes were *PKD1, PKD2, COL4A5, COL4A4*, and *HNF1B* accounting for 62.6% (*n* = 62/99) of the resolved cases (Fig. 1C, D). Across both age groups, the most frequently identified gene was *PKD1*, found in 42.6% of adults (*n* = 29/68) and 29.0% of children (*n* = 9/31). Among adults, this was followed by *PKD2* (13.2%, *n* = 9), and *COL4A5* (10.3%, *n* = 7), while in the pediatric group, *HNF1B* (9.7%, *n* = 3) and *COL4A4* (6.5%, *n* = 2) were the next most common genes (Fig. 1C, D). Among patients with cystic kidney diseases, *PKD1* was the most frequently affected gene (64.4%, *n* = 38/59), followed by *PKD2* (15.3%, *n* = 9) and *IFT140* (5.1%, *n* = 3). *COL4A5* (47.1%, *n* = 8/17) and *COL4A4* (23.5%, *n* = 4) were most causative in the glomerulopathies category. No pathogenic *MUC1* variants were identified despite targeted analysis across the entire cohort.

In 7.4% (*n* = 19/256) of the cases, variants of uncertain significance (VUS, ACMG class 3) were detected (Supplementary Table S4). Interestingly, the detection of VUS was more than double in pediatric patients at 12.3% (*n* = 10/81) compared to 5.1% (*n* = 9/175) in adults (*P* = .069; *). Secondary findings were detected in 1.2% of patients (*n* = 3/256, Supplementary Table S5).

### Age at genetic diagnosis varies across kidney disease groups

We analyzed age at first diagnosis across disease groups to identify age-specific diagnostic patterns. The number of tested individuals decreased with increasing age, with 61.3% of tests performed between 0 and 40 years (*n* = 157/256, Fig. 1E, Supplementary Table S6). In all CAKUT cases, a genetic diagnosis was established during childhood or adolescence, most frequently before the age of 10 years (10/26), and none were diagnosed beyond age 20 years (0/15). Detection rates in this group remained low across all age groups (Fig. 1E, Supplementary Table S6). Tubulopathies were most frequently diagnosed in early life (0–20 years), with declining diagnostic frequency in older individuals (Fig. 1E, Supplementary Table S6). Glomerulopathies displayed a bimodal pattern, with a prominent peak in adulthood (40–60 years) and a secondary peak in childhood. In contrast, cystic kidney diseases were predominantly diagnosed between ages 40 and 60 years and demonstrated consistently high genetic detection rates across all age groups (Fig. 1E, Supplementary Table S6).

### Impact of family history, extrarenal manifestations, proteinuria, and hypertension on genetic detection rates

**Figure 2:**
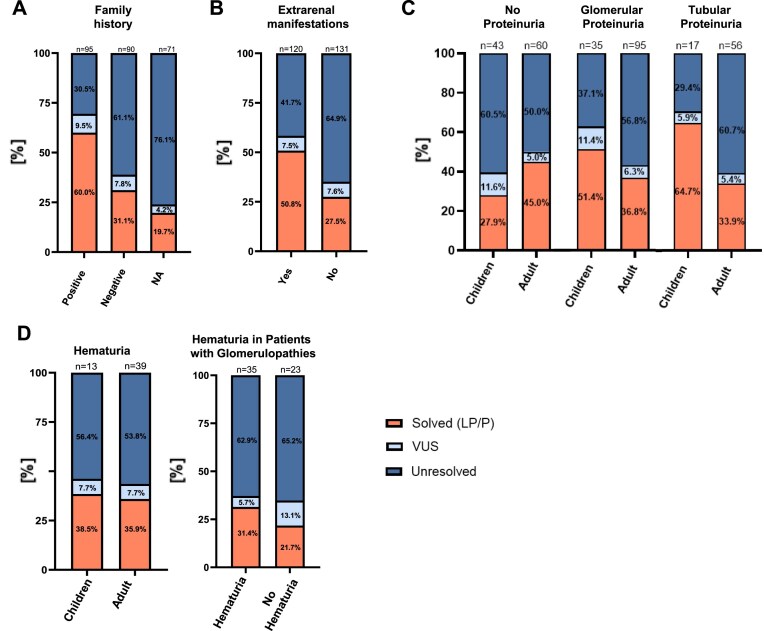
Impact of extrarenal features and urinary findings on diagnostic yield. (A) Diagnostic yield by family history in pediatric and adult patients. (B) Comparison of diagnostic yield in patients with extrarenal manifestations versus isolated kidney disease. (C) Diagnostic yield in children and adults stratified by tubular and glomerular proteinuria. (D) Solving rate in patients with hematuria, overall and by glomerulopathy status.

In patients with suspected IKD, 37.1% (*n* = 95/256) reported a positive family history. The diagnostic yield was significantly higher in this group (60.0%, *n* = 57/95), compared to those with a negative family history (31.1%, *n* = 28/90, *P* = .00 002, ***) and those without family history information (19.7%, *n* = 14/71, *P* ≤ .0001, ^****^, Fig. 2A).

Patients presenting with extrarenal manifestations had a higher diagnostic yield (50.8%, *n* = 61/120) than those with isolated kidney disease (27.5%, *n* = 36/131; Fig. 2B). To assess the impact of proteinuria, patients were stratified by subtype (none, glomerular, or tubular). In children, the detection rate was significantly higher in those with tubular (64.7%, *n* = 11/17; *P* = .0168) and glomerular proteinuria (51.4%, *n* = 18/35; *P* = 0.0388) compared with children without proteinuria (27.9%, *n* = 12/43; Fig. 2C). In adults, the diagnostic yield did not differ across proteinuria subtypes (Fig. 2C). The presence of hematuria was associated with a similar diagnostic yield in children and adults (36.5%, *n* = 19/52; Fig. 2D). Among patients with glomerulopathies, the detection rate was slightly higher in those with hematuria (31.4%, *n* = 11/35) compared to those without hematuria (21.7%, *n* = 5/23, *P* = .5493, ns, Fig. 2D).

In the overall cohort, multivariable logistic regression identified hypertension (OR 3.44, 95% CI 1.66–7.35) and extrarenal manifestations (OR 2.05, 95% CI 1.08–3.96) as independent positive predictors of genetic diagnosis. A positive family history (reference; OR 1.00) was associated with higher odds compared to negative (OR 0.30, 95% CI 0.15–0.61) and unknown (OR 0.13, 95% CI 0.05–0.31) family history, while all other variables were not significantly associated (Supplementary Table S7). Including ADPKD status as a covariate identified ADPKD as the strongest independent predictor of achieving a genetic diagnosis (OR 8.29, 95% CI 3.77–19.22; Supplementary Table S8). A positive family history remained independently associated with genetic diagnosis (Supplementary Table S8). Arterial hypertension also remained independently associated with genetic diagnosis (OR 2.98, 95% CI 1.35–6.80), indicating an increased likelihood of a positive genetic finding irrespective of ADPKD status. In contrast, extrarenal manifestations were no longer significantly associated with genetic diagnosis after adjustment for ADPKD (OR 1.70, 95% CI 0.84–3.45, Supplementary Table S8). Age, baseline eGFR, sex, proteinuria subtype, hematuria, and recurrent urinary tract infections were not significantly associated with genetic diagnosis (Supplementary Tables S7, S8). In the pediatric subgroup, tubular proteinuria was a significant independent predictor of genetic diagnosis (OR 4.17, 95% CI 1.21–16.09; Supplementary Table S7), remaining associated even after adjustment for tubulopathies.

Kaplan-Meier analysis showed significantly reduced kidney survival in patients with severe proteinuria (>300 mg/g) compared to those with no or mild proteinuria (<300 mg/g; *P* < .0001, ***; Supplementary Table S9). The median age at KF was ∼50 years with proteinuria versus 68 years in those without. In contrast, kidney survival did not differ significantly according to the presence of extrarenal manifestations. The median age at 50% KF was 58.0 years in patients with extrarenal features and 62.0 years in those without (*P* = .7131, ns; Supplementary Table S9). To assess whether this effect was driven by cystic kidney disease, we analyzed kidney survival by disease category. Except for cystic disease (50% KF at 66.0 years), extrarenal manifestations were associated with earlier KF across all other groups: glomerulopathies (46.0 years), tubulopathies (46.0 years), and CAKUT (34.0 years), Supplementary Table S9).

### Earlier kidney failure in genetically solved male patients

Among genetically solved patients, 50% reached KF by 60.0 years of age compared to 64.0 years in genetically unsolved individuals (*P* = .7632, ns, Supplementary Fig. S1A). We then compared kidney survival outcomes among the most commonly affected genes: *PKD1, PKD2, COL4A5, COL4A4*, and *HNFF1B*. ADPKD was associated with the most favorable kidney prognosis. Individuals with *PKD2* variants progressed to KF significantly later, with 50% reaching KF at 69.5 years compared to 52 years in *PKD1* mutation carriers (*; *P* = .0485). *COL4A5*-associated AS genotypes are characterized by an accelerated decline in kidney function, with 50% of patients progressing to KF by 32 years of age (Supplementary Fig. S1B). In contrast, none of the patients carrying a *COL4A4* variant developed KF (*n* = 4). Among the three patients harboring *HNF1B* variants, one progressed to KF at an early age of 5.0 years (Supplementary Fig. S1B). Comparing kidney survival among different disease categories, patients with tubulopathies and cystic kidney diseases (50% reaching KF at 68.0 years) showed the most favorable kidney outcome. Patients with glomerulopathies exhibited the poorest kidney survival, with 50% experiencing KF by the age of 49.0 years. CAKUT revealed a median KF at 59.0 years (Supplementary Fig. S1C). Interestingly, across all disease groups, genetically solved cases showed poorer kidney survival (Supplementary Fig. S1D).

**Figure 3:**
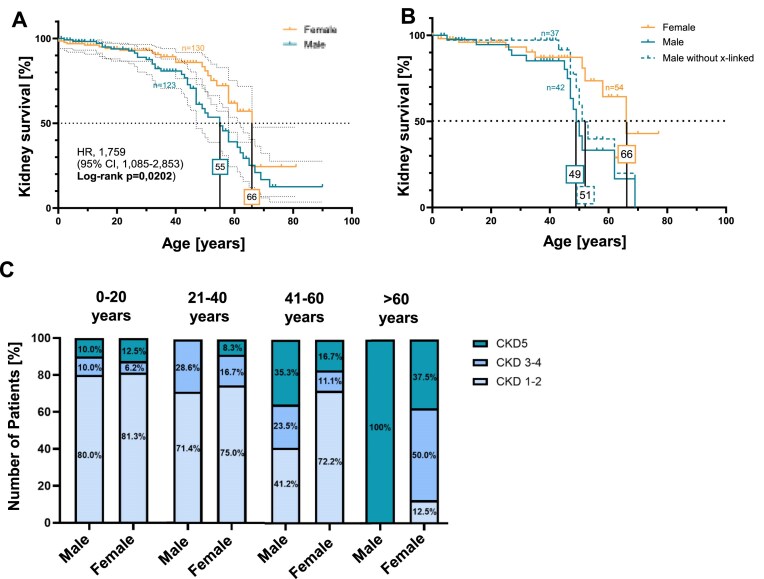
Sex differences in kidney survival and CKD stage distribution (A) Kidney survival comparison between female and male patients in the total cohort independent of genetic resolution. (B) Kidney survival in male and female patients (50% KF 49.0 vs. 66.0 years; *P* = .0216). The blue dotted line shows kidney survival in male patients after excluding X-linked cases. The difference compared with genetically affected women is attenuated and no longer statistically significant (51.0 vs. 66.0 years; *P* = .1670). (C) CKD stages stratified by age and sex after excluding males with X-linked disorders.

Data on sex differences in IKD and KF are scarce; we therefore compared kidney survival by sex. In the total cohort, females tended to reach the 50% kidney survival endpoint later (66.0 years) than males (55.0 years, *P* = .0202, *, Fig. 3A). When stratifying kidney survival by sex and genetic diagnosis, men with a confirmed genetic cause tend to show earlier KF than unsolved male cases (50% KF at 49.0 vs. 58.0 years; *P* = .5208, ns). In females, no difference was observed between solved and unsolved cases (50% KF at 66.0 years in both; *P* = .5549, ns). Kidney survival significantly differed between solved males and females (50% KF 49.0 vs. 66.0 years; *P* = .0216, *, Fig. 3B). However, after excluding X-linked cases, this sex difference was attenuated and no longer statistically significant (51.0 vs. 66.0 years; *P* = .1670, Fig. 3B). Across the genetically solved cohort, we analyzed CKD stage distribution by age group after excluding males with X-linked disorders (Fig. 3C). CKD stage distributions were similar between sexes in younger patients (0–40 years). However, from age 40 onward, particularly after 60 years, female patients tended to remain in lower CKD stages compared with males (Fig. 3C).

### Copy number variants in pediatric patients

CNVs represent an important cause of kidney disease in pediatric cohorts, especially in CAKUT and syndromic cases [25, 26]. Our cohort revealed disease-causing CNVs in 13.9% of children with CAKUT (*n* = 5/36). Interestingly, in adults we did not detect a CNV. In addition to two 17q12 deletions observed in patients with *HNF1B*-associated disease, other pathogenic CNVs included complex rearrangements such as trisomy 10p, partial trisomy 13 mosaicism, and large deletions or duplications involving *SIX1* and *IFT140* (Supplementary Table S1). We identified a novel complex CNV comprising a monoallelic 1 Mb deletion at 6p25, a monoallelic 17 Mb duplication spanning 6p25.3–6p22.3, and a monoallelic 5 Mb duplication of 9p24; the detailed phenotypic and genetic characterization of this case is provided in the Supplementary Material (Supplementary Fig. S2, Supplementary Text 1).

## DISCUSSION

In this cohort of 256 patients with suspected IKD, genetic testing identified pathogenic variants in 38.7% of cases. Multiple studies, along with our findings, demonstrate that a core set of 10 or fewer genes, including *PKD1, PKD2, COL4A5, COL4A4*, and *HNF1B*, collectively account for ∼60% of diagnosed cases across various kidney disease phenotypes [12]. Prior to next-generation sequencing (NGS), sequential single-gene testing was laborious and limited in scope [27]. The introduction of panel sequencing significantly improved diagnostic yields in IKDs [28, 29] and ES has since expanded the number of known monogenic disease genes to over 600 [30]. In our cohort, ES was applied in the majority of pediatric patients, starting with virtual panels and expanding to the full exome when no pathogenic variant was identified [31]. This approach is particularly effective for unspecific, atypical or overlapping phenotypes, such as AS, nephrotic syndrome, ciliopathies, and CAKUT, where targeted panels may miss relevant variants [31]. In our cohort, the proportion of VUS was higher in pediatric patients, who predominantly underwent ES, compared to adults (Fisher’s exact test *P* = .069). While this observation may reflect the broader genomic coverage of ES compared to targeted panel approaches, it should be interpreted cautiously given the limited sample size and lack of statistical significance [16, 17]. Despite targeted analysis, no pathogenic *MUC1* variants were identified, which is consistent with the very low prevalence of ADTKD-*MUC1*, estimated at ∼0.7–4 cases per million in population-based studies [32].

A key strength of our study is the inclusion of both pediatric and adult patients, a design shared by only two other studies [12, 13]. Although IKDs often manifest in childhood, the overall diagnostic yield was similar between pediatric and adult patients (38.7%). This can be explained by the differing distribution and genetic detectability of disease subtypes across age groups. In the pediatric cohort, the high proportion of CAKUT cases–typically associated with lower detection rates–reduced overall yield. In contrast, adults more frequently presented with cystic kidney diseases and glomerulopathies (particularly AS), which are linked to higher genetic resolution. However, lower yields in adult tubulopathies and CKDx balanced these differences. Consequently, the similar overall diagnostic yield in both groups reflects a compensation of subtype-specific differences between age groups. Extrarenal manifestations were associated with genetic diagnosis in the unadjusted analysis but lost significance after adjustment for ADPKD, indicating that this effect was largely driven by this subgroup. Nevertheless, such features remain clinically relevant and should prompt consideration of genetic testing, particularly in patients with suspected ADPKD. Arterial hypertension remained independently associated with genetic diagnosis after adjustment for ADPKD. However, as hypertension is not an established predictor of genetic diagnostic yield, this finding should be interpreted cautiously. The relatively small cohort size, inclusion of pediatric patients, where hypertension is less common and more likely to indicate underlying genetic disease, and potential residual confounding may have influenced this association. In addition, hypertension was assessed retrospectively at the time of study inclusion rather than at initial clinical presentation. Therefore, it remains unclear whether hypertension preceded or followed the decline in kidney function, and it may primarily reflect disease severity rather than serving as an early diagnostic predictor.

Our data shows that tubular proteinuria is a significant predictor of obtaining a genetic diagnosis in children with kidney disease. To test whether this effect may only be driven by patients with tubulopathies, we performed an adjusted multivariable analysis. In this model, tubular proteinuria remained significantly associated with genetic diagnosis, while tubulopathy itself was not (OR 1.23, 95% CI 0.12–11.98), suggesting that the association with tubular proteinuria is not fully explained by the presence of tubulopathies, although residual confounding and limited sample size cannot be excluded. Genetic testing, being noninvasive and diagnostically superior to biopsy, is particularly valuable in conditions such as Fanconi syndrome and nephronophthisis, where histology is often nonspecific and standard therapies like ACE inhibitors offer limited benefit [33–35]. Our findings support the use of genetic testing as a first-line diagnostic approach in pediatric patients with tubular proteinuria to ensure early and accurate diagnosis.

Several studies have suggested sex differences in CKD progression, particularly in younger individuals [36–38]. However, these studies mainly included patients with diabetic nephropathy, hypertension-induced CKD, glomerulopathies, or immune-mediated diseases, and data on sex differences in genetic kidney disease cohorts remain scarce [36–38]. In our total cohort of patients with suspected IKD, we similarly observed that women had significantly better kidney survival than men. Sex-related differences were also evident within genetically solved cases, where women showed better kidney survival than men, indicating that sex influences disease course in IKD. One major driver of this pattern is the more severe progression of X-linked disorders, which typically lead to earlier and more aggressive kidney involvement in male patients. After excluding X-linked cases, the observed sex difference persisted only as a nonsignificant trend, likely reflecting reduced statistical power following subgroup restriction. Moreover, this residual trend may, at least in part, be influenced by the ADPKD sub-cohort, in which sex-specific differences in disease progression have been described, rather than representing a uniform feature across all IKDs [39, 40]. Another factor discussed in the literature is the potential nephroprotective effect of estrogen. Estrogen has been shown to attenuate glomerulosclerosis and reduce kidney injury [41, 42]. Conversely, estrogen deficiency––particularly after early oophorectomy––has been associated with an increased risk of CKD [43–45]. Consequently, male patients with IKD may be at risk for a more severe disease course. This underscores the importance of thorough genetic characterization, including precise variant identification and determination of X-linked inheritance, for accurate prognostic counseling. In addition, male patients should be specifically educated about their potentially higher risk of progression and the critical importance of strict control of modifiable risk factors, particularly proteinuria and hypertension, as well as adherence to close clinical follow-up.

CNVs are frequently identified in renal disease, particularly in CAKUT, ciliopathies, and tubulopathies [26, 46, 47]. In CAKUT, particularly in cases with renal hypodysplasia or agenesis, CNVs are reported in up to 10%–14% of patients [48, 49]. A novel CNV in our cohort involved three genes previously linked to CAKUT, yet it remains unclear which of them drives the phenotype. This illustrates a general challenge of CNV interpretation: single-gene attribution is often not possible, but CNVs can broaden diagnostic yield and point to new candidate pathways [49]. In our cohort, no CNVs were detected in adults. One contributing factor may be that CNV-rich phenotypes (e.g. CAKUT, early onset CKD, and ciliopathies) predominantly become apparent in pediatric and young-adult populations, and CNV analysis is more frequently pursued when syndromic or neurodevelopmental features are suspected [48–50]. In adult patients undergoing panel-based testing, CNV detection was limited to genes included in the respective panel, and CNVs affecting other genomic regions potentially relevant to the phenotype may therefore have remained undetected. Consequently, broader implementation of CNV analysis in adult cohorts may improve the diagnostic yield in genetic kidney disease.

This study provides a comprehensive genetic and clinical analysis of pediatric and adult IKD patients, demonstrating the value of a phenotype-driven, tiered diagnostic strategy. In children, tubular proteinuria shows a high predictive value for a genetic diagnosis (OR 4.17) and represents a clinically relevant red flag to guide early genetic testing. Poorer outcomes in genetically confirmed cases, particularly in men, underscore the importance of integrating genetic diagnostics with detailed clinical characterization in routine care.

## Supplementary Material

sfag160_Supplemental_Files

## Data Availability

Anonymized datasets underlying this study can be obtained from the corresponding author upon reasonable request.
