# Supplementary material for: Clinical impact of genetic testing in inherited kidney diseases
Source: Clin Kidney J. 2026 May 19;19(7):sfag160. doi: 10.1093/ckj/sfag160 (PMC13320241; doi:10.1093/ckj/sfag160)
Supplement: sfag160_Supplemental_Files [file sfag160_supplemental_files.zip › 1812 supplementary figures.docx]

**
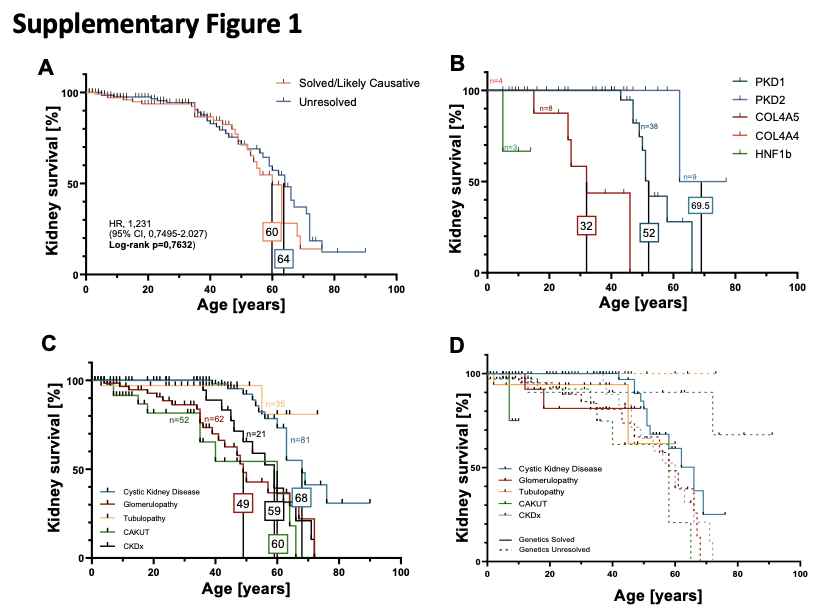
**

**Supplementary Figure 1: Kidney survival across genetic and disease categories. (A)Genetically solved and unsolved patients showed similar kidney survival (50% KF at 60.0 vs. 64.0 years; p = 0.7632, ns). (B) Among the most frequent genes, PKD2 variant carriers reached KF later than PKD1 carriers (69.5 vs. 52.0 years; p = 0.0485), while COL4A5 showed the earliest KF (32 years). (C) Tubulopathies and cystic kidney diseases had the most favorable outcome (50% KF at 68.0 years), followed by CAKUT (59.0 years), whereas glomerulopathies showed the poorest kidney survival (49.0 years). (D) Across disease groups, genetically solved cases exhibited poorer kidney survival.**

**
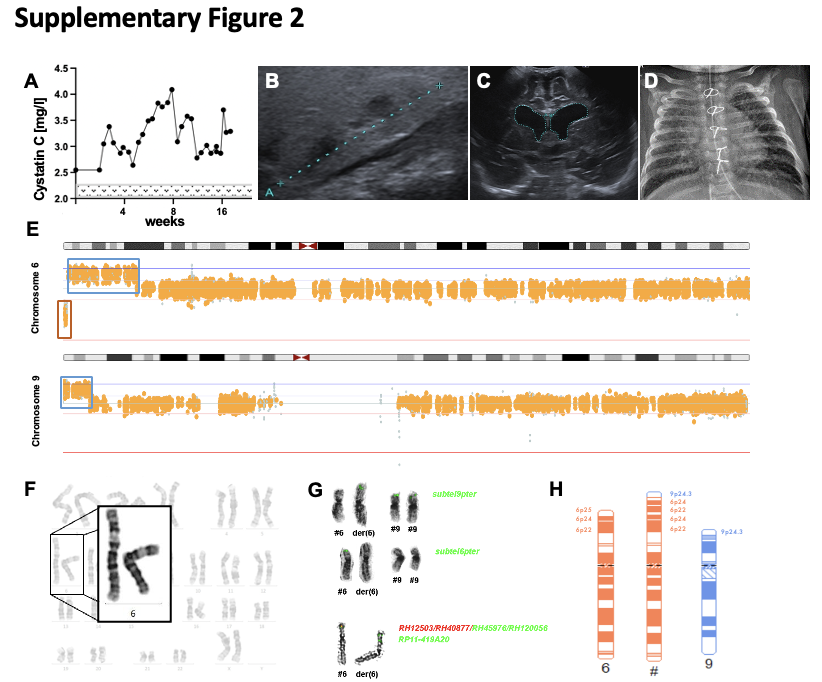
**

**Supplementary Figure 2: Clinical and genetic findings in a newborn with syndromic CAKUT. (A–D)** Clinical presentation including chronic kidney disease, kidney dysplasia, brain anomalies, and cardiac malformation. **(E)** Exome-based CNV analysis highlighting chromosomal deletions and duplications. **(F)** Karyotype analysis using GTG banding. **(G)** FISH analysis demonstrating complex rearrangements involving chromosomes 6 and 9. **(H)** Ideogram illustrating the derivative chromosome 6 with structural rearrangements.
