# Supplementary material for: Clinical impact of genetic testing in inherited kidney diseases
Source: Clin Kidney J. 2026 May 19;19(7):sfag160. doi: 10.1093/ckj/sfag160 (PMC13320241; doi:10.1093/ckj/sfag160)
Supplement: sfag160_Supplemental_Files [file sfag160_supplemental_files.zip › Supplementary Tables_final.docx]

**SUPPLEMENTARY FILES**

**SUPPLEMENTARY TABLE 1: Diagnostic Overview**

|  | **Exome sequencing** | **Panel-based sequencing** | **Targeted Testing of a Familial Variant** |
| --- | --- | --- | --- |
| **Adults** | 42 | 131 | 2 |
| **Children** | 66 | 13 | 2 |

**SUPPLEMENTARY TABLE 2: Genotypic and phenotypic information of patients with genetically solved kidney diseases**

Abbreviations: f female; m male; Het. heterozygous; Hom. Homozygous; Hem. hemizygous; P pathogenic; LP likely pathogenic; NA not available; NK not known; ADPKD autosomal dominant polycystic kidney disease; TMA thrombotic microangiopathy; DM diabetes mellitus; ADTKD autosomal dominant tubulointerstitial kidney disease; PLD polycystic liver disease; RTA renal tubular acidosis; FHHNC familial hypomagnesemia with hypercalciuria and nephrocalcinosis; CKD chronic kidney disease; FSGS focal segmental glomerulosclerosis; aHUS atypical hemolytic-uremic syndrome. *complex chromosomal rearrangement of chromosome 6 with one deletion spanning *DUSP22, IRF4,* and *EXOC2* genes, an intrachromosomal insertion affecting *SLC35B3*, *DEK*, *SIRT5,* and more then 80 other genes. Furthermore, one duplication at the beginning of chromosome 9 and an intrachromosomal insertion affecting *GLIS3, DOCK8, SMARCA2*, and 24 other genes.

| **ID (sex)** | **Kidney Phenotype** | **Extrarenal Phenotype** | **Family History / Consanguinity (yes/no)** | **Gene** | **NM** | **C Change** | **P Change** | **Zygosity** | **ACMG** | **Reference** |
| --- | --- | --- | --- | --- | --- | --- | --- | --- | --- | --- |
| g233 (f) | Polycystic kidney degeneration | Liver cysts | positive / no | *PKD1* | NM_001009944.3 | c.12691C>T | p.(Gln4231*) | Het. | P | Stekrova 2009 BMC Med Genet |
| g195 (f) | ADPKD | Hepatomegaly | negative / no | *PKD1* | NM_001009944.3 | c.10966del | p.(Leu3656Trpfs*28) | Het. | P | Audrézet 2012 Hum Mutat |
| g108 (f) | TMA | Short stature, craniofacial abnormalities | negative / no | *CFI* | NM_000204.5 | c.1291G>T | p.(Ala431Ser) | Het. | LP | NK |
| g487 (m) | Renal glucosuria | No | NA | *SLC5A2* | NM_003041.4 | c.885+5G>A | p.? | Hom. | P | Santer 2003 JASN |
| g198 (f) | Hematuria, kidney cysts | Scoliosis, liver cysts | positive / no | *COL4A3* | NM_000091.4 | c.1892G>T | p.(Gly631Val) | Het. | P | Weber 2016 Pediatr Nephrol |
| g447 (f) | Diabetic-hypertensive nephropathy | DM Type I | negative / no | *NOTCH2* | NM_025508.4 | c.7119del | p.(His2374Ilefs*42) | Het. | LP | NK |
| g639 (m) | Bilateral kidney cysts | Liver cysts | positive / no | *PRKCSH* | NM_001289104.2 | c.300C>A | p.(Cys100*) | Het. | P | ClinVar |
| g403 (f) | ADPKD | Cerebral medullary lesions, left leg paresis, liver cysts | positive / no | *PKD1* | NM_001009944.3 | c.856_862del | p.(Gly287*) | Het. | P | O`Brien 2012 J Pedriatr Gastroenterol Nutr |
| g619 (f) | Kidney cysts | Epilepsy, autism, intellectual disability, LAM | negative / no | *TSC2/PKD1* | NM_000548.5 / NM_000296.3 | c.5261_*1179delinsGGT / c.12851_*1280delinsACC, | p.? / p.? | Het/Het. | P | NK |
| g132 (f) | ADTKD | No | positive / no | *UMOD* | NM_003361.4 | c.335G>A | p.Cys112Tyr | Het. | LP | Utami 2015 Clin Exp Nephrol |
| g653 (m) | ADPKD, bilateral kidney cysts | Liver cysts | positive / no | *PKD1* | NM_001009944.3 | c.6696_6699del | p.(Phe2232Leufs*9) | Het. | P | ClinVar |
| g114 (f) | Nephronophthisis | Cardiomyopathy, Retinal dystrophy | negative / no | *CEP290* | NM_025114.4 | c.2279_2280del, / c.5493del | p.(Phe760*) / p.(Ala1832Profs*19) | Compound Het. | P/P | NK / Brancati 2007 Am J Hum Genet |
| g343 (f) | Liver dominant phenotype with two single kidney cysts | Multiple sklerosis, liver cysts, liver transplantation | positive / no | *PKD2* | NM_000297.4 | c.423_430dup | p.(Tyr144Leufs*92) | Het. | P | NK |
| g393 (m) | Cystinuria | NA | NA | *SLC3A1* | NM_000341.4 | c.1400T>C, / c.1871C>T, | p.(Met467Thr) / p.(Thr624Ile) | Het./Het. | P/P | Calonge 1994 Nat Genet / NK |
| g203 (f) | ADPKD | Marfan-Syndrome | positive / no | *PKD1* | NM_001009944.3 | c.7570_7572del | p.(Glu2524del) | Het. | LP | NK |
| g204 (f) | ADPKD/PLD | Liver cysts | negative / no | *PRKCSH* | NM_001289104.2 | c.310G>C | p.(Asp104His) | Het. | P | NK |
| g486 (m) | ADPKD | Liver cysts | positive / no | *PKD1* | NM_001009944.3 | c.6994_7000del | p.(Ala2332Trpfs*7) | Het. | P | Phakdeekitcharoen 2000 Kidney Int |
| g601 (m) | ADPKD | Liver cysts | negative / no | *PKD2* | NM_000297.4 | c.2062A>T | p.(Lys688*) | Het. | P | NK |
| g213 (f) | ADPKD | Liver cysts | positive / no | *PKD1* | NM_001009944.3 | c.9530dup | p.(Ser3178*) | Het. | P | Neumann 2013 NDT |
| g338 (m) | ADPKD | No | positive / no | *PKD1* | NM_001009944.3 | c.6994_7000dup | p.(Val2334Glyfs*88) | Het. | P | Rossetti 2001 Am J Hum Genet |
| g366 (m) | ADPKD, unilateral nephrectomy due to huge kidney cyst | Liver cysts, cardiomyopathy | positive / no | *PKD1* | NM_001009944.3 | c.8016+2T>A | p.? | Het. | P | NK |
| g209 (f) | ADPKD | Liver cysts | negative / no | *PKD1* | NM_001009944.3 | c.9038C>T | p.(Ser3013Phe) | Het. | LP | ClinVar |
| g210 (m) | Cystic kidney | Skeletal anomalies | positive / no | *PKD1* | NM_001009944.3 | c.1295C>A | p.(Ala432Glu) | Het. | LP | ClinVar |
| g660 (m) | Nephrolithiasis | No | positive / no | *SLC7A9* | NM_014270.5 | c.604+2T>C | p.? | Het. | P | Font-Llitjos 2005 J Med Genet |
| g220 (f) | Cystic kidneys | Liver cysts | positive / no | *PKD2* | NM_000297.4 | c.1458C>G | p.(Tyr486*) | Het. | P | Lindemann 2023 Kidney Int Rep |
| g222 (f) | ADPKD | Liver cysts, pancreatic cysts | positive / no | *PKD1* | NM_001009944.3 | c.10261_10264del | p.(Pro3421Thrfs*51) | Het. | P | ClinVar |
| g223 (m) | ADPKD | Liver cysts | NA | *PKD1* | NM_001009944.3 | c.8324_8325insA | p.(Leu2776Alafs*46) | Het. | LP | ClinVar |
| g404 (f) | Alport Syndrome, pyelonephritis, high growth | No | positive / no | *COL4A5* | NM_033380.3 | c.3545G>T | p.Gly1182Val | Het. | P | Weber 2016 Pediatr Nephrol |
| g228 (m) | Cystic kidneys | No | negative / no | *PKD1* | NM_001009944.3 | c.8017-2_8017-1del | p.? | Het. | P | Rossetti 2001 Am J Hum Genet |
| g229 (f) | Alport Syndrome | Deafness | positive / no | *COL4A4* | NM_000092.5 | c.4470del | p.(Tyr1491Ilefs*61) | Het. | P | Mallett 2017 Kidney Int |
| g235 (m) | ADPKD | Liver cysts | positive / no | *PKD2* | NM_000297.4 | c.1094+1G>A | p.? | Het. | P | Chung 2006 Clin Genet |
| g239 (m) | Cystic kidneys | Liver cysts | positive / no | *PKD1* | NM_001009944.3 | c.9400A>G | p.(Thr3134Ala) | Het. | LP | Yu 2022 NPJ Genom Med |
| g241 (m) | ADPKD | No | positive / no | *PKD1* | NM_001009944.3 | c.1021dup | p.(Ala341Glyfs*30) | Het. | P | NK |
| g243 (f) | Cystic kidneys | Liver cysts | negative / no | *PKD1* | NM_001009944.3 | c.7704-1G>C | p.? | Het. | P | NK |
| g245 (m) | ADPKD | Idiopathic Parkinson's syndrome, incomplete tetraparesis | NA | *PKD2* | NM_000297.4 | c.2435_2436del | p.(Ser812*) | Het. | P | Hwang 2016 JASN |
| g250 (f) | ADPKD | No | positive / no | *PKD1* | NM_001009944.3 | c.755_771del | p.(Pro252Hisfs*3) | Het. | P | Neumann 2013 NDT |
| g253 (f) | Alport Syndrome, persistent microhematuria | No | negative / no | *COL4A4* | NM_000092.5 | c.3044G>A | p.(Gly1015Glu) | Het. | P | Papazachariou 2017 Clin Genet |
| g255 (f) | Kidney cysts | Liver cysts | positive / no | *PKD1* | NM_001009944.3 | c.9397+1_9398-1)_(10167+1_10168-1) | p.? | Het. | LP | NK |
| g257 (f) | Nephrocalcinosis, nephrolithiasis, medullary sponge kidney, RTA | No | positive / no | *ATP6V0A4* | NM_020632.2 | c.1631C>T | p. (Ser544Leu) | Het. | LP | Mori 2017 Clin Exp Nephrol |
| g262 (f) | Kidney cysts | Liver cysts | NA | *PKD1* | NM_001009944.3 | c.2857T>G | p.(Tyr953Asp) | Het. | LP | ClinVar |
| g263 (f) | ADPKD | Liver cysts | positive / no | *PKD1* | NM_001009944.3 | c.8447T>C | p.(Leu2816Pro) | Het. | LP | Rossetti 2001 Am J Hum Genet |
| g268 (f) | Proteinuria, hematuria | No | NA | *COL4A5* | NM_033380.3 | c.1871G>A | p.(Gly624Asp) | Het. | LP | Martin 1998 JASN |
| g269 (f) | Cystic kidneys | No | positive / no | *PKD2* | NM_000297.4 | c.2614C>T | p.(Arg872*) | Het. | P | Reynolds 1999 JASN |
| g273 (m) | Alport Syndrome | Multiple sklerosis | NA | *COL4A5* | NM_033380.3 | c.3508G>A | p.(Gly1170Ser) | Het. | P | Inoue 1999 Am J Kidney Dis |
| g276 (w) | ADPKD | Liver cysts | negative / no | *PKD1* | NM_001009944.3 | c.1723-2A>C | p.? | Het. | P | Audrézet 2012 Hum Mutat |
| g278 (m) | Nephrolithiasis, FHHNC | Coronary three-vessel disease | NA | *CLDN16* | NM_006580.4 | c.243G>T \| c.415G>A | p.(Leu81Phe) \| p.(Ala139Thr) | Compound Het. | P | Weber 2000 Eur J Hum Genet / Weber 2001 JASN |
| g279 (m) | ADTKD | No | positive / no | *UMOD* | NM_003361.4 | c.707C>G | p.(Pro236Arg) | Het. | P | Bernascone 2006 Traffic |
| g280 (f) | ADPKD | Mitral and tricuspid valve insufficiency, liver cysts, sigmadiverticulosis, cerebral aneurysm | positive / no | *PKD1* | NM_001009944.3 | c.9298C>T \| c.9049T>G | p.(Gln3100*) \| p.(Tyr3017Asp) | Compound Het. | LP | Neumann 2013 NDT / NK |
| g281 (m) | ADPKD | No | positive / no | *PKD2* | NM_000297.4 | c.637C>T | p.(Arg213*) | Het. | P | Robinson 2012 BMC Nephrol |
| g283 (m) | Primary FSGS | No | negative / no | *NPHS2* | NM_014625.4 | c.413G>A \| c.868G>A | p.(Arg138Gln) \| p.(Val290Met) | Compound Het. | P | Boute 2000 Nat Genet / Karle 2002 JASN |
| g289 (m) | Cystic kidneys | Liver cysts | positive / no | *PKD1* | NM_001009944.3 | c.3586_3687 del | p.(Thr1196Glyfs*14) | Het. | LP | NK |
| g300 (m) | Cystic kidneys | Liver cysts | positive / no | *PKD2* | NM_000297.4 | c.973C>T | p.(Arg325*) | Het. | P | Rossetti 2007 JASN |
| g302 (f) | Cystic kidneys | No | positive / no | *PKD1* | NM_001009944.3 | c.2857T>G | p.(Tyr953Asp) | Het. | LP | ClinVar |
| g307 (f) | ADPKD/ADPLD | Liver cysts | positive / no | *PKD2* | NM_000297.4 | c.1094+1G>A | p.? | Het. | P | Chung 2006 Clin Genet |
| g311 (m) | Tubulopathy with hypokalemia, Gitelman Syndrome | No | NA | *SLC12A3* | NM_001126108.2 | c.247C>T, \| c.1067C>T | p.(Arg83Trp) \| p.(Ala356Val) | Compound Het. | P | Vargas-Poussou 2011 JASN / Ji 2008 Nat Genet |
| g312 (m) | Alport Syndrome | Hearing loss, tricuspid valve insufficiency | positive / no | *COL4A5* | NM_033380.3 | c.1226G>T | p.Gly409Val | Het. | P | ClinVar |
| g320 (w) | APOL1-associated chronic kidney disease, hypertension | Sickle cell anemia | negative / no | *APOL1* | NM_003661.4 | c.1164_1169del \| c.(1024A>G;c.1152T>G) | p.(Asn388_Tyr389del) \| p.(Ser342Gly;Ile384Met) | Compound Het | LP | Genovese 2010 Science / Genovese 2010 Science / Genovese 2010 Science |
| g402 (m) | Alport Syndrome | Hearing loss | positive / no | *COL4A5* | NM_033380.3 | c.3097G>T | p.(Gly1033Cys) | Hem. | P | ClinVar |
| g396 (f) | pRTA | Short stature, cataract, secondary glaucoma with glaucoma papilla, astigmatism myopicus compositus | positive / yes | *SLC4A4* | NM_001098484.3 | c.1170G>C | p.(Arg390Ser) | Hom. | VUS (*in silicio* consistently pathogenic) | Godbole, 2023 Case Report |
| g401 (m) | ADPKD | No | positive / no | *PKD1* | NM_001009944.3 | c.10050+143_10499+770del | p.? | Het. | P | NK |
| g435 (m) | Cystic kidneys | No | positive / no | *IFT140* | NM_014714.4 | c.1565G>A | p.(Gly522Glu) | Het. | LP | Perrault 2012 Am J Hum Genet |
| g436 (m) | Bilateral cystic kidneys | Pyloric stenosis, left-sided herniotomy, prolactinome | NA | *PKD1* | NM_001009944.3 | c.11488_11489del | p.(Ser3830Profs*130) | Het. | P | ClinVar |
| g461 (f) | ADPKD | Liver cysts | NA | *PKHD1* | NM_138694.4 | c.9148G>T, \| c.6992T>A, | p.(Gly3050Cys) \| p.(Ile2331Lys) | Het. | VUS/P | NK / Ward 2002 Nat Genet |
| g467 (m) | Cystic kidneys | No | positive / no | *IFT140* | NM_014714.4 | c.2399+1G>T |  | Het. | P | Perrault 2012 Am J Hum Genet |
| g468 (f) | Alport Syndrome | Corneal curvature, strabismus | positive / no | *COL4A5* | NM_033380.3 | c.1871G>A | p.(Gly624Asp | Het. | P | Martin 1998 JASN |
| g548 (f) | ADPKD | Liver cysts | NA | *PKD1* | NM_001009944.3 | c.2945_3295+222del |  | Het. | P | NK |
| g549 (m) | Nephrocalcinosis, CKD of unknown origin | No | positive / no | *KCNJ1* | NM_153766.3 | c.89G>A | p.(Cys30Tyr) | Hom. | P | ClinVar |
| g701 (m) | Alport Syndrome | Hearing loss | NA | *COL4A5* | NM_033380.3 | c.3453dup | p.(Gly1152Argfs*32) | Het. | P | ClinVar |
| C002 (w) | Ehlers-Danlos Syndrome | Scoliosis, hearing loss, developmental disorder | positive / yes | *CHST14* | NM_130468.4 | c.145del | p.Val49fs | Hom. | P | Dundar 2009 Am J Hum Genet |
| C006 (w) | FSGS | No | positive / no | *TRIM8* | NM_030912.3 | c.1213_1219delinsCAGTACGG | p.Thr405Glnfs*5 | Het. | P | NK |
| C012 (fetus) | Cystic kidneys | No | positive / no | *PKHD1* | NM_138694.3 | c.664A>G, \| c. 7916C>A, | p.I222V \| p.Ser2839* | Het. / Het. | P/P | Ward 2002 Nat Genet/ Denamur 2010 Kidney Int |
| C020 (f) | Hematuria | No | positive / no | *COL4A4* | NM_000092.5 | c.5048G>A | p.Cys1683Tyr | Het. | LP | Weber 2016 Pediatr Nephrol |
| C023 (m) | Cystic kidneys | No | positive / no | *PKD1 \| PRKCSH* | NM_001009944.3 \| NM_001289104.2 | c.1723-1G>C, \| c.205G>A, | p.? \| p.Ala69Thr | Het. | P | NK/ NK |
| C030 (m) | Bilateral kidney dysplasia | No | positive / no | *HNF1b* | NM_000458.4 | c.443C>T | p.Ser148Leu | Het. | P | Edghill 2006 J Med Genet |
| C031 (f) | Knobloch Syndrome type 1 | Opticus dysplasia | positive / no | *COL18A1* | NM_001379500.1 | c.3514_3515del_CT | p.Leu1172VAlfs*72 | Hom. | P | NK |
| C032 (m) | Polyuria, hypokalemia, hypercalciuria | No | positive / yes | *CTNS* | NM_004937.3 | c.18_21del | p.(Thr7Phefs*7) | Hom. | P | Town 1998 Nat Genet |
| C034 (f) | Bilateral polycystic kidney dysplasia | Dystrophy | positive / no | *HNF1b* | NM_000458.4 | Microdeletion Chr. 17q12 |  | Het. | P | Testard 2022 J Med Genet |
| C038 (f) | Cystic kidneys | No | NA | *PKD1* | NM_001009944.3 | c.5995G>A | p.Gly1999Ser | Het. | LP | Rossetti 2007 J Am Soc Nephrol |
| C039 (f) | ADPKD | No | positive / no | *PKD1* | NM_001009944.3 | c.10719dupA | p.Gly3574Argfs*53 | Het. | LP | NK |
| C042 (f) | Renal agenesis on the right, pyelonephritis | Muscular hypotony, developmental delay, hearing loss | negative / no | *Trisomie 10p (47XX+del(10)(q11))* |  | Trisomie 10p (47XX+del(10)(q11)) |  | Het. | P | NK |
| C045 (f) | ADPKD | No | positive / no | *PKD1* | NM_001009944.3 | c.9458_9484inv | p.His3153_Arg3162delinsArgTrpLysAlaLeuSerProSerArgCys | Het. | LP | ClinVar |
| C046 (f) | BOR Syndrome | Short stature, developmental delay, autistic traits | negative / no | *SIX1 + big deletion in region 5p13.3* | NM_005982 | c.1A>C | p.0? | Het. | P | NK |
| C047 (f) | Cystic kidneys | Short stature, ADHD, developmental delay | positive / no | *Deletion 17q12 + SCN8A* | NM_001330260.2 | Deletion 17q12 SCN8A: c.2685A>C, | p.(Gln895His) | Het. | P | Testard 2022 J Med Genet \| NK |
| C051 (m) | ADPKD | No | negative / no | *PKD1* | NM_001009944.3 | c.8269_8288delinsAA | p.Ser2757_Leu2763delinsAsn | Het. | LP | NK |
| C052 (f) | Alport Syndrome | Small hearing loss | positive / no | *COL4A4* | NM_000092.5 | c.994G>A | p.Gly332Arg | Het. | LP | ClinVar |
| C057 (f) | Bilateral medullary sponge kidney | Developmental delay, epilepsy, nystagmus, cataract | negative / no | *COL4A1* | NM_001845.6 | c.1573G>A, c.2078G>A | p.(Gly525Arg) \| p.(Gly693Glu) | Compound Het. | LP | NK \|  Livingston (2011) Neuropediatrics |
| C064 (m) | Multicystic kidney dysplasia | No | negative / no | *PKD1* | NM_001009944.3 | c.8049C>A | p.Cys2683* | Het. | P | Nielsen 2021 Eur J Med Genet |
| C066 (f) | Cystic kidneys | No | negative / no | *PKD1* | NM_001009944.3 | c.8299C>T | p.Arg2767Csy | Het. | P | Garcia-Gonzalez (2007) Mol Genet Metab |
| C068 (f) | Cystic kidneys | No | negative / no | *PKD1* | NM_001009944.3 | c.3308T>G | p.Leu1103Arg | Het. | LP | ClinVar |
| C069 (m) | aHUS | No | negative / no | *CFH \| CD46* | NM_000186.4 \| NM_172351.3 | c.157C>T, \| c.286+2T>G, | p.Arg53Cys \| p.? | Het. | P/P | Fakhouri 2010 JASN\| Fremeaux-Bacchi 2006 JASN |
| C071 (m) | Alport Syndrome | Hearing loss | positive / no | *COL4A5* | NM_033380.3 | c.904A>T | p.Lys302* | Hem. | P | NK |
| C079 (m) | ADPKD | No | positive / no | *PKD1* | NM_001009944.3 | c.11326_11329delTTCA | p.Phe3776Alafs*49 | Het. | P | NK |
| C080 (f) | Kidney insufficiency | Ocular involvement, polysplenia | positive / no | *IFT140* | NM_014714 | Duplication Exons 27-30, and c.1039C>T | p.Arg347* | Compound Het. | Dup LP, FS P | Dup: Lemire 2024 Am J Hum Genet  FS: Xu 2020 Br J Ophthalmol |
| C083 (f) | Cystic kidney dysplasia | Inguinal hernie, retinopathy | negative / no | *HNF1B/TCF2* | NM_000458.4 | Deletion Exons 1-9 |  | Het. | P | Raile 2009 J Clin Endocrinol Metab |
| C090 (f) | Kidney dysplasia | Pulmonary vein anomaly, vena cava stenosis | negative / no | one big deletion, an insertion and two duplications affecting over 100 protein coding genes |  | chr6: 6p25.3p25.3: g.(pter_60501) (1111587_1311883)del chr6p25.3p22.3(656994x11111588_18226523x3,18236633x2), NC_000006.11:g(656995_1111588)-(18226523_18236623)ins, 9p24.3p24.1(pter,15859_5467912x35522497x2), NC_000009.11:g.(pter_15859)-(5467912_5522496)ins * |  | Het. | P | NK |
| C095 (m) | Multicystic kidney dysplasia, obstructed urinary flow | Developmental delay | negative / no | *DYRK1A* | NM_001347721.2 | c.475G>C | p.(Gly159Arg) | Het. | P | ClinVar |
| C101 (f) | Bohring-Opitz-Syndrome | Combined developmental delay, hearing loss | negative / no | *ASXL* | NM_015338.6 | c.4048C>T | p.(Gln1350*) | Het. | P | ClinVar |
| C102 (f) | Nephronophthisis | Dystrophy | negative / yes | *INVS* | NM_014425.5 | c.2786+2T>C | p.? | Hom. | P | Wang 2021 Front Genet |
| C103 (m) | Kidney cysts | Developmental delay | negative / no | Partial Trisomy 13 mos 47, XY,+13[19]/46, XY[11], 63% mosaic |  | Partial Trisomy 13 mos 47, XY,+13[19]/46, XY[11], 63% mosaic |  |  | P | NK |

**SUPPLEMENTARY TABLE 3: Comparison of Clinical Characteristics Between the Solved and Unresolved Cohorts**

Abbreviations: CKD chronic kidney disease; KTx kidney transplantation; CAKUT congenital anomalies of the kidney and urinary tract; NA not available, IQR interquartile range.

|  | | **Solved cohort** | **Unresolved cohort** |
| --- | --- | --- | --- |
| **Age (years)** | | 40.0 (IQR 19.3-50.0) | 36.0 (IQR 13.8-49.3) |
| **Age at genetic testing (years)** | | 36.0 (IQR 16.0-47.0) | 34.0 (IQR 12.0-46.5) |
| **Sex** | | m: 43.4% (n=43)  f: 55.6% (n=55) | m: 51.6% (n=81)  f: 48.4% (n=76) |
| **Family history** | | Positive: 58.6% (n=58)  Negative: 27.2% (n=27)  NA: 14.2% (n=14) | Positive: 24.2% (n=38)  Negative: 39.5% (n=62)  NA: 36.3% (n=57) |
| **Extrarenal manifestations** | | Yes: 61.6% (n=61)  No: 36.4% (n=36)  NA: 2.0% (n=2) | Yes: 37.6% (n=59)  No: 60.5% (n=95)  NA: 1.9% (n=3) |
| **Kidney function** | CKD stage 1-2 | 56.6% (n=56) | 54.1% (n=85) |
|  | CKD stage 3-4 | 16.2% (n=16) | 19.1% (n=30) |
|  | CKD stage 5, KF | 24.2% (n=24) | 27.8% (n=42) |
|  | KTx | 7.0% (n=7) | 9.6% (n=15) |
| **Urinary findings** | Hematuria (y/n) | y: 19.2% (n=19)  n: 74.7% (n=74)  NA: 6.1% (n=6) | y: 21.0% (n=33)  n: 69.4% (n=109)  NA: 9.6% (n=15) |
|  | Glomerular proteinuria | 52.5% (n=52) | 47.1% (n=74) |
|  | Tubular proteinuria | 30.3% (n=30) | 28.7% (n=43) |
| **Clinical disease groups** | Cystic Kidney Diseases | 59.6% (n=59) | 14.6% (n=23) |
|  | Glomerulopathies | 17.2% (n=17) | 31.3% (n=49) |
|  | Tubulopathies | 12.1% (n=12) | 14.6% (n=23) |
|  | CAKUT | 10.1% (n=10) | 26.8% (n=42) |
|  | CKD of Unknown Origin (CKDx) |  | 12.0% (n=21) |

**SUPPLEMENTARY TABLE 4: Genotypic and Phenotypic Information of Patients with Variants of Unknown Significance (VUS)**

Abbreviations: f female; m male; Het. heterozygous; hom. Homozygous; NA not available; NK not known; ADPKD autosomal dominant polycystic kidney disease; TMA thrombotic microangiopathy; CKD chronic kidney disease; LUTO lower urinary tract obstruction; VUR vesicouretral reflux

| **ID (sex)** | **Kidney Phenotype** | **Extrarenal Phenotype** | **Family**  **History**  **/ Consanguinity (yes/no)** | **Gene** | **NM** | **C Change** | **P Change** | **Zygosity** | **ACMG** | **Reference** |
| --- | --- | --- | --- | --- | --- | --- | --- | --- | --- | --- |
| g458 (f) | ADPKD | Liver cysts | positive / no | *PKD2* | NM_000297.4 | c.709+5G>A | p.? | Het. | VUS | Kimura (2023) Biomolecules |
| g199 (m) | Microangiopathy, CKD3 | Retinopathy, liver cirrhosis, several apoplex | NA | *TREX1* | NM_033629.6 | c.703dup | p.(Val235Glyfs*6) | Het. | VUS | Richards 2007 Nat Genet |
| g430 (m) | ADPKD | Liver cysts, liver cirrhosis | positive / no | *PKD1* | NM_001009944.2 | c.11660C>G | p.(Pro3887Arg) | Het. | VUS | Carrera et al., 2016 |
| g232 (m) | FSGS | No | negative / no | *WT1* | NM_024426.6 | c.434T>G | p.(Ile145Ser) | Het. | VUS | NK |
| g230 (f) | Microhematuria | Alpha-thalassemia major | positive / no | *COL4A3* | NM_000091.5 | c.1637C>T | p.(Pro546Leu) | Het. | VUS | Rao 2019 Clin Genet |
| g258 (f) | Nephrolithiasis bilateral | No | NA | *SLC34A3* | NM_001177316.2 | c.1093+41_1094-15del85 | p.? | Het. | VUS | Ichikawa 2006 J Clin Endocrinol Metab |
| g270 (f) | TMA | Hypertensive heart disease | negative / no | *CD46* | NM_172350 | c245G>A | p.(ARg82Gln) | Het. | VUS | NK |
| g288 (f) | Cystic kidneys | Liver cysts | positive / no | *PKD1* | NM_001009944.3 | c.3162-3C>G | p.? | Het. | VUS | ClinVar |
| g587 (f) | ADPKD | No | NA | *PKD1* | NM_001009944.3 | c.12464T>C | p.(Phe4155Ser) | Het. | VUS | ClinVar |
| C001 (m) | Bartter-Syndrome Type 3 | Dystrophy, short stature | positive / yes | *CLCNKB* | NM_000085.5 | Complete deletion |  | Hom. | VUS | Simon (1997) Nat Genet |
| C013 (f) | ADPKD | No | positive / no | *PKD1* | NM_001009944.3 | c. 2879G>T | p.Gly960Val | Het. | VUS | NK |
| C015 (f) | Multicystic kidney dysplasia right | Renofacial dysplasia | positive / no | *GREB1L* | NM_001142966.3 | c.968G>A | p.(Gly323Glu) | Het. | VUS | ClinVar |
| C035 (m) | Kidney insufficiency, dystopic single kidney on the left, VUR | No | negative / no | *ROBO2* | NM_001128929.3 | c.340G>A | p.Gly114Arg | Het. | VUS | NK |
| C053 (m) | Microhematuria | No | positive / no | *COL4A3* | NM_000091.5 | c.1637C>T | p.Pro546Leu | Het. | VUS | Rao 2019 Clin Genet |
| C063 (m) | Bilateral kidney dysplasia, kidney insufficiency, LUTO | No | positive / no | *SLIT2* | NM_004787.4 | c.3899A>G | p.Asn1300Ser | Het. | VUS | NK |
| C082 (f) | Glomerulopathy | No | negative / no | *COL4A4* | NM_000092.5 | c.1099+5G>A | p.? | Het. | VUS | ClinVar |
| C085 (f) | Steroid-resistant nephrotic syndrome | No | negative / no | *TRPC6* | NM_004621.6 | c.2409+8A>T | p.? | Het. | VUS | NK |
| C087 (m) | Nephrolithiasis, obstructed urinary flow | Short stature | negative / no | *SLC3A1* | NM_000341.4 | Duplikation Exon 5-9 \| c.1134C>T | p.= a.e. | Het. | VUS | Bleyer 2022 Am J Nephrol \| NK |
| C096 (m) | Kidney cysts | No | negative / no | *PKD1* | NM_001009944.3 | c.216-8C>G | p.? | Het. | VUS | Cornec-Le Gall 2016 JASN |

**SUPPLEMENTARY TABLE 5: Genotypic and Phenotypic Information of Patients with Incidental Findings**

Abbreviations: f female; m male; Het. heterozygous; LP likely pathogenic; NA not available; NK not known; TMA thrombotic microangiopathy.

| **ID (sex)** | **Kidney Phenotype** | **Extrarenal Phenotype** | **Family**  **History**  **/ Consanguinity (yes/no)** | **Gene** | **NM** | **c Change** | **P Change** | **Zygosity** | **ACMG** | **Reference** |
| --- | --- | --- | --- | --- | --- | --- | --- | --- | --- | --- |
| g287 (m) | Podocytopathy | MEN 1 Syndrome | NA | *MEN1* | NM_001370259.2 | c.524T>C | p.Leu175Pro | Het. | LP | Caswell 2022 Genome Med |
| g303 (m) | TMA | Retinitis Pigmentosa | positive / no | *ABCA4* | NM_000350.3 | c.514G>A c.3962C>T | p.Gly172Ser p.Ser1321Phe | Comp. Het. | LP/VUS | Jaakson (2003) Hum Mutat \| NK |
| C022 (f) | Bilateral renal dysplasia nephrolithiasis | Developmental delay, strabismus | negative / no | *NF1* | NM_001042492.3 | c.339del | p.Arg1132Valfs*10 | Het. | LP | NK |

**SUPPLEMENTARY TABLE 6: Diagnostic Yields**

Abbreviations: CAKUT congenital anomalies of the kidney and urinary tract.

| **Diagnostic Yield** | **CAKUT** | **Cystic kidney diseases** | **Tubulopathies** | **Glomerulopathies** |
| --- | --- | --- | --- | --- |
| 0-20 | 27% (n=10/37) | 61% (n=14/23) | 33% (n=2/6) | 32% (n=6/19) |
| 20-40 | 0% (n=0/9) | 75% (n=15/20) | 38% (n=6/16) | 17% (n=5/29) |
| 40-60 | 0% (n=0/3) | 83% (n=24/29) | 40% (n=4/10) | 50% (n=6/12) |
| 60-80 | 0% (n=0/3) | 60% (n=6/10) | 0% (n=0/3) | 0% (n=0/6) |

## SUPPLEMENTARY TABLE 7: Multivariable Logistic Regression Predicting Genetic Diagnosis in the Overall Cohort (Without Adjustment for ADPKD) and Pediatric Subgroup

| Variable | β (Estimate) | Standard Error | 95% CI (β) | Odds Ratio (expβ) | 95% CI (OR) |
| --- | --- | --- | --- | --- | --- |
| Age | -0.0046 | 0.0094 | -0.0232 – 0.0138 | 0.995 | 0.977 – 1.014 |
| eGFR | 0.0034 | 0.0052 | -0.0068 – 0.0136 | 1.003 | 0.993 – 1.014 |
| Sex (male) | -0.1841 | 0.3319 | -0.8370 – 0.4687 | 0.832 | 0.433 – 1.598 |
| Family history (negative vs positive) | -1.1930 | 0.3645 | -1.9230 – -0.4890 | 0.303 | 0.146 – 0.613 |
| Family history (unknown vs positive) | -2.0540 | 0.4778 | -3.0480 – -1.1590 | 0.128 | 0.047 – 0.314 |
| Extrarenal manifestations (no vs yes) | 0.7190 | 0.3303 | 0.07569 – 1.375 | 2.052 | 1.079 – 3.955 |
| Glomerular proteinuria (no vs reference) | 0.1460 | 0.4250 | -0.6894 – 0.9841 | 1.157 | 0.5019 – 2.675 |
| Hematuria (yes) | -0.1880 | 0.4058 | -0.9968 – 0.6025 | 0.829 | 0.369 – 1.827 |
| Hypertension (no vs yes) | 1.235 | 0.3773 | 0.5089 – 1.995 | 3.439 | 1.663 – 7.349 |
| Recurrent UTI (yes) | 0.3071 | 0.5588 | -0.8052 – 1.409 | 1.359 | 0.4470 – 4.093 |
| Tubular proteinuria (no vs reference) | -0.08382 | 0.4806 | -1.033 – 0.8606 | 0.9196 | 0.3560 – 2.365 |
| Pediatric Subgroup Analysis: Tubular Proteinuria Adjusted for Tubulopathies (selected variables shown) | | | | | |
| Tubular proteinuria (children, yes vs no) | 1.428 | 0.6493 | 0.1883 – 2.778 | 4.169 | 1.207 – 16.09 |
| Tubulopathy (yes vs no) | 0.2103 | 1.108 | -2.119 – 2.484 | 1.234 | 0.1201 – 11.98 |

**SUPPLEMENTARY TABLE 8: Multivariable Logistic Regression Predicting Genetic Diagnosis in the Overall Cohort (Adjusted for ADPKD)**

| Variable | β (Estimate) | Standard Error | 95% CI (β) | Odds Ratio (expβ) | 95% CI (OR) |
| --- | --- | --- | --- | --- | --- |
| Age | -0.01786 | 0.01075 | -0.03944 – 0.002913 | 0.9823 | 0.9613 – 1.003 |
| eGFR | 0.001730 | 0.005566 | -0.009302 – 0.01268 | 1.002 | 0.9907 – 1.013 |
| Sex (male vs female) | -0.4051 | 0.3646 | -1.129 – 0.3066 | 0.6669 | 0.3232 – 1.359 |
| Family history (negative vs positive) | -1.167 | 0.4004 | -1.970 – -0.3928 | 0.3114 | 0.1395 – 0.6751 |
| Family history (unknown vs positive) | -1.548 | 0.5161 | -2.608 – -0.5684 | 0.2127 | 0.07366 – 0.5664 |
| Extrarenal manifestations (yes vs no) | 0.5287 | 0.3580 | -0.1724 – 1.237 | 1.697 | 0.8416 – 3.445 |
| Glomerular proteinuria (yes vs no) | 0.2587 | 0.4618 | -0.6453 – 1.174 | 1.295 | 0.5245 – 3.236 |
| Tubular proteinuria (yes vs no) | 0.3740 | 0.5360 | -0.6726 – 1.440 | 1.454 | 0.5104 – 4.222 |
| Hematuria (yes vs no) | 0.09634 | 0.4389 | -0.7741 – 0.9551 | 1.101 | 0.4611 – 2.599 |
| Hypertension (yes vs no) | 1.092 | 0.4105 | 0.2994 – 1.917 | 2.980 | 1.349 – 6.798 |
| Recurrent UTI (yes vs no) | 0.02489 | 0.5998 | -1.161 – 1.214 | 1.025 | 0.3133 – 3.368 |
| ADPKD (yes vs no) | 2.115 | 0.4136 | 1.327 – 2.956 | 8.288 | 3.770 – 19.22 |

**SUPPLEMENTARY TABLE 9: Kidney Survival in Patients with Proteinuria or Extrarenal Manifestations**

Abbreviations: KF kidney failure; ns not significant; CAKUT congenital anomalies of the kidney and urinary tract; CKDx chronic kidney disease of unknown origin.

| **Kidney survival with vs. without proteinuria separated by genetic result** | | | | | | | |
| --- | --- | --- | --- | --- | --- | --- | --- |
|  | **Proteinuria ≥ level A3** | |  | **No proteinuria < level A3** | | **log-rank test** |  |
|  | **n** | **Age: 50% at KF** |  | **n** | **Age: 50% at KF** | **p (curve comparison)** | **Significance** |
| **GENETICS SOLVED** | 33 | 49 |  | 85 | < 50% experienced KF | <0,0001 | **** |
| **GENETICS UNRESOLVED** | 37 | 50 |  | 96 | 68 | <0,0001 | **** |
| **Kidney survival with vs. without extrarenal manifestations** | | | | | | | |
|  | **Extrarenal manifestations** | |  | **No extrarenal manifestations** | | **log-rank test** |  |
|  | **n** | **Age: 50% at KF** |  | **n** | **Age: 50% at KF** | **p (curve comparison)** | **Significance** |
|  | 117 | 62 |  | 129 | 58 | 0,7131 | ns |
| **Kidney survival with vs. without extrarenal manifestations separated by disease groups** | | | | | | | |
|  | **Extrarenal manifestations** | |  | **No extrarenal manifestations** | | **log-rank test** |  |
|  | **n** | **Age: 50% at KF** |  | **n** | **Age: 50% at KF** | **p (curve comparison)** | **Significance** |
| **CYSTIC** | 49 | 66 |  | 31 | 69 | 0,9694 | ns |
| **GLOMERULOPATHY** | 26 | 46 |  | 33 | 47 | 0,7905 | ns |
| **TUBULOPATHY** | 12 | 46 |  | 21 | < 50% experienced KF | 0,0322 | * |
| **CAKUT** | 26 | 34 |  | 26 | 58 | 0,2098 | ns |
| **CKDx** | 4 | 46 |  | 17 | 54 | 0,7071 | ns |

**SUPPLEMENTARY Text 1: A Novel Complex CNV in a Patient with CAKUT**

We identified a novel CNV including the chromosomal regions 6p25 and 9p24 in a newborn who presented with kidney dysfunction (eGFR 18 mL/min/1.73 m², KDIGO stage 4; Pat. ID C0090, **Supplementary Figures 4A**), bilateral kidney dysplasia **(Supplementary Figure 4B)**, facial dysmorphism, and enlarged intracerebral fluid spaces at birth **(Supplementary Figure 4C)**. The patient also suffered from chronic respiratory insufficiency requiring permanent invasive ventilation **(Supplementary Figure 4D)**. Echocardiography revealed a total anomalous pulmonary venous connection, requiring surgical anastomosis to the left atrium. Postoperatively, the patient developed severe restenosis within 4.5 weeks. As repeated dilation posed high risk with limited benefit, palliative care was initiated. The patient died in hospital at two months of age.

Coverage-based CNV analysis from exome sequencing revealed a complex chromosomal rearrangement comprising a monoallelic 1 Mb deletion at 6p25, a monoallelic 17 Mb duplication spanning 6p25.3 to 6p22.3, and a monoallelic 5 Mb duplication of 9p24 **(Supplementary Figure 4E)**. Analysis of alternative allele frequencies of inherited variants indicated that all three CNVs were located on the paternal allele and had occurred *de novo*, with matching pathogenic entries found in DECIPHER and ClinVar. Within the 6p25.3–6p22.3 duplicated region, two established CAKUT-associated genes were identified: *FOXC1^1^* and *TFAP2A.^2^* The 9p24.3–p24.1 duplication encompassed *GLIS3*, implicated in cystic dysplasia.^3^

Karyotyping and FISH confirmed a complex structural rearrangement of chromosome 6, involving a terminal deletion from 6pter to 6p25.3. At the breakpoint, the telomeric segment of chromosome 9 (9pter to 9p24.1) was inserted, followed by a direct duplication of 6p25.3 to 6p22.3, and a continuation of chromosome 6 from 6p25.3 to the q-terminal end **(Supplementary Figures 4F-H)**.

1. Wu CHW, Mann N, Nakayama M, et al. Phenotype expansion of heterozygous FOXC1 pathogenic variants toward involvement of congenital anomalies of the kidneys and urinary tract (CAKUT). *Genet Med*. 2020;22(10):1673. doi:10.1038/S41436-020-0844-Z

2. Milunsky JM, Maher TM, Zhao G, et al. Genotype-phenotype analysis of the branchio-oculo-facial syndrome. *Am J Med Genet A*. 2011;155(1):22-32. doi:10.1002/AJMG.A.33783,

3. Dimitri P, Habeb AM, Garbuz F, et al. Expanding the Clinical Spectrum Associated With GLIS3 Mutations. *J Clin Endocrinol Metab*. 2015;100(10):E1362. doi:10.1210/JC.2015-1827
